# Prolonged Influenza Virus Shedding and Emergence of Antiviral Resistance in Immunocompromised Patients and Ferrets

**DOI:** 10.1371/journal.ppat.1003343

**Published:** 2013-05-23

**Authors:** Erhard van der Vries, Koert J. Stittelaar, Geert van Amerongen, Edwin J. B. Veldhuis Kroeze, Leon de Waal, Pieter L. A. Fraaij, Roland J. Meesters, Theo M. Luider, Bart van der Nagel, Birgit Koch, Arnold G. Vulto, Martin Schutten, Albert D. M. E. Osterhaus

**Affiliations:** 1 Department of Virology, ErasmusMC, Rotterdam, The Netherlands; 2 Viroclinics Biosciences B.V., Rotterdam, The Netherlands; 3 Department of Paediatrics, ErasmusMC-Sophia, Rotterdam, The Netherlands; 4 Department of Neurology, ErasmusMC, Rotterdam, The Netherlands; 5 Department of Hospital Pharmacy, ErasmusMC, Rotterdam, The Netherlands; Paul-Ehrlich-Institut, Germany

## Abstract

Immunocompromised individuals tend to suffer from influenza longer with more serious complications than otherwise healthy patients. Little is known about the impact of prolonged infection and the efficacy of antiviral therapy in these patients. Among all 189 influenza A virus infected immunocompromised patients admitted to ErasmusMC, 71 were hospitalized, since the start of the 2009 H1N1 pandemic. We identified 11 (15%) cases with prolonged 2009 pandemic virus replication (longer than 14 days), despite antiviral therapy. In 5 out of these 11 (45%) cases oseltamivir resistant H275Y viruses emerged. Given the inherent difficulties in studying antiviral efficacy in immunocompromised patients, we have infected immunocompromised ferrets with either wild-type, or oseltamivir-resistant (H275Y) 2009 pandemic virus. All ferrets showed prolonged virus shedding. In wild-type virus infected animals treated with oseltamivir, H275Y resistant variants emerged within a week after infection. Unexpectedly, oseltamivir therapy still proved to be partially protective in animals infected with resistant virus. Immunocompromised ferrets offer an attractive alternative to study efficacy of novel antiviral therapies.

## Author Summary

Immunocompromised patients, such as transplant recipients on immune suppressive therapy, are a substantial and gradually expanding patient group. Upon influenza virus infection, these patients clear the virus less efficiently and are more likely to develop severe pneumonia than immunocompetent individuals. Existing antiviral strategies are far from satisfactory for this patient group, as they show limited effectiveness with frequent emergence of antiviral resistance. For ethical and practical reasons antiviral efficacy studies are hard to conduct in these patients. Therefore, we developed an immunocompromised ferret, mimicking an immune suppressive regimen used for solid organ transplant recipients. Upon infection with 2009 pandemic influenza A/H1N1 virus these animals, like immunocompromised patients, develop severe respiratory disease with prolonged virus excretion. Interestingly, all immunocompromised ferrets on oseltamivir therapy excreted oseltamivir resistant viruses (H275Y) within one week after start of treatment. Furthermore, high dose oseltamivir therapy still proved to be partially effective against these oseltamivir resistant viruses. These immunocompromised ferrets provide a useful tool in the development of novel antiviral approaches for immunocompromised patients suffering from influenza.

## Introduction

During the first 12 months of the 2009 influenza A/H1N1 virus (pH1N1) pandemic an estimated 284,000 patients died and hospitalization rates were considerably higher than for seasonal influenza [1]. Although many severe cases were observed in otherwise healthy patients under 50 years of age, most fatal cases during this pandemic were patients belonging to the traditional high risk groups for developing severe disease, like very young children, the elderly and chronically ill patients [2]. In these patients, which in most cases have sub-optimal immune responses, influenza viruses often persists longer and tend to spread more readily into the lower respiratory tract [3], [4], [5], [6]. These observations are in contrast to those in otherwise healthy patients younger than 65 years, for which influenza usually remains a self-limiting upper respiratory tract infection [7], [8].

It has been recognized that every winter season a significant number of immunocompromised patients are admitted to a hospital with influenza [9], [10]. For example, of the total 335 influenza A virus infected patients being diagnosed upon admission to ErasmusMC - a tertiary university hospital - between August 2009 and July 2012, 113 (34%) had an underlying condition that classified them as being immunocompromised [11]. Since immunocompromised patients are more likely to acquire influenza [12], [13], showing relatively high influenza-associated mortality [4], [14], [15], [16], [17], [18], [19], [20], [21], [22], [23], [24], effective antiviral prophylaxis and treatment protocols are of crucial importance for these patients.

Unfortunately, present antiviral strategies are merely based on clinical trials conducted in otherwise healthy patients [25], since randomized clinical trials in immunocompromised patients are, for both ethical and practical reasons, difficult to perform. Furthermore, the degree and cause of a patient's immunocompromised state is variable and consequently, clinical outcome of infection may vary accordingly.

Although antiviral therapy has a documented positive effect on clinical outcome in immunocompromised patients [3], [26], [27], [28], current antiviral strategies are far from satisfying. This may be explained not only by the lack of evidence based strategies adjusted for immunocompromised patients [4], [28], but also by the oral and inhaled administration routes which complicate administration in very young and critically ill patients [29], [30], . Furthermore, since physicians may not consider the diagnosis influenza initially, antiviral therapy is often initiated beyond 48 hours [34], and accompanied by the emergence of an oseltamivir resistant virus [35].

We investigated the incidence of prolonged virus shedding and emergence of antiviral resistance by studying the course of infection of the immunocompromised patients infected with pH1N1 virus treated in our university hospital. These phenomena were studied in more detail in immunocompromised ferrets experimentally infected with pH1N1 virus, that closely mimic immunocompromised patients with influenza. These ferrets all showed prolonged virus shedding and emergence of antiviral resistance. Unexpectedly, the group of immunocompromised ferrets treated with an oseltamivir dose equivalent to a 450 mg dose (twice daily) in humans, had a higher survival rate than similarly untreated animals when infected with an oseltamivir resistant virus.

## Results

### Prolonged shedding and resistance development in immunocompromised patients

**Figure 1:**
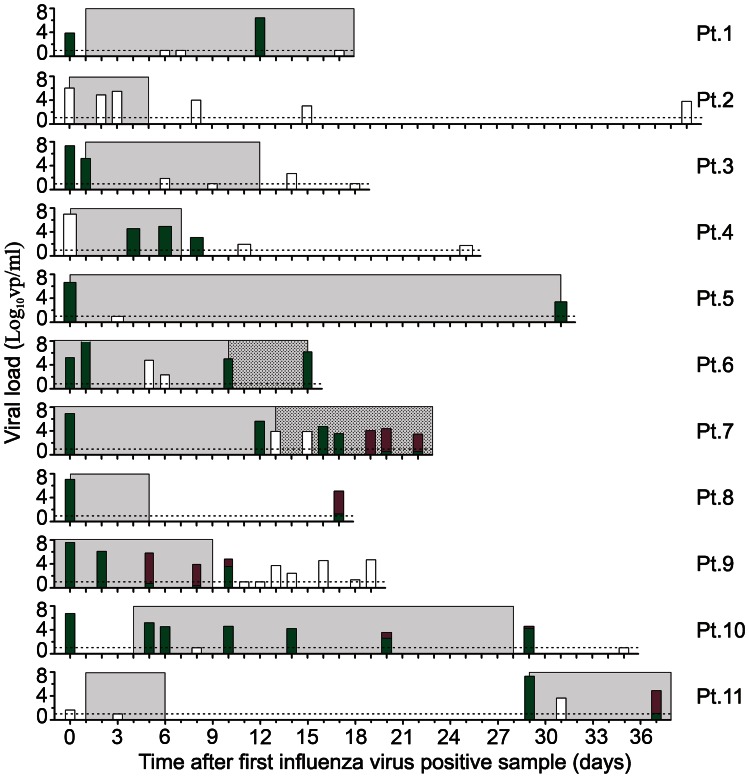
Viral load, antiviral therapy and resistance detection in immunocompromised patients hospitalized with a prolonged pH1N1 virus infection. From 11 immunocompromised patients the viral load in respiratory specimens obtained during the courses of illness are shown in bars. Patients 1, 2 and 10 were solid organ transplant patients. Patients 3, 4, 5, 7, 8, 9 and 11 were cancer patients on chemotherapy. Patient 6 was treated for cerebral vasculitis. The dotted line in each figure indicates the lower limit of detection of the influenza A virus RT-qPCR detection assay. Data are presented as the number of virus particles per ml. Bar colours indicate the absence (green) or presence (magenta) of the H275Y oseltamivir resistance mutation as detected by RT-PCR. If both genotypes were detected in a sample, the proportion is stacked. Bars are coloured white for those respiratory samples in which the H275Y genotype could not be determined or in cases when genotyping was not performed. The duration of oseltamivir monotherapy and oseltamivir/zanamivir combination therapy is indicated, respectively, by blue and dotted blue shading.

We quantified prolonged virus replication and resistance development in immunocompromised patients retrospectively, who were infected between August 2009 and July 2012, and hospitalized in our tertiary hospital with influenza A virus. Among the 189 RT-qPCR confirmed influenza A virus infected patients (median age = 22.3, range = 0–81), 71 (38%) patients were classified as being immunocompromised (Table 1). These patients were either cancer patients on chemotherapy (CC), solid organ transplant (SOT) or patients with an auto-immune disease on immune suppression, HIV-infected patients or patients with another cause of compromised immune status. From 37 (52%) patients, no follow up samples were taken (physician's choice) and 18 (25%) patients had cleared the virus within 14 days. Of the immunocompromised patients from whom follow up was available, 11 (15%) had a pH1N1 virus infection and were shedding virus for more than 14 days, despite receiving oseltamivir or oseltamivir/zanamivir therapy (Figure 1). Prolonged replication of influenza A/H3N2 virus was found in 5 (7%) cases (data not shown). In 5 (7%) of the immunocompromised patients with an influenza pH1N1 virus infection the oseltamivir resistance mutation H275Y in the neuraminidase was detected by RT-PCR during oseltamivir mono or oseltamivir/zanamivir combination therapy.

**Table 1:** Immune status and antiviral therapy of 2009 pandemic influenza A virus infected patients hospitalized in our tertiary hospital between August 2009 and July 2012.

Total hospitalized influenza A virus infected patients^a^^,^ b ^,^ c		n = 189 (%)
Immunocompromised patients		71 (38)
**Cause of compromised immune status (n = 71)**		
Cancer chemotherapy	41 (58)	
Solid organ transplant recipients	12 (17)	
Auto-immune disease	7 (10)	
HIV/AIDS^d^	3 (4)	
Other^e^	8 (11)	
**Antiviral therapy**	*Immunocompromised*	*All Hospitalized*
Any	54 (76)	137 (72)
Oseltamivir monotherapy	44 (62)	117 (62)
Zanamivir monotherapy	1 (1)	1 (<1)
Combination therapy	9 (13)	19 (10)
None	17 (24)	50 (26)
Unknown^f^	None	2 (1)

a Influenza A virus was detected by an influenza A virus real-time quantitative polymerase-chain-reaction (RT-qPCR) detection assay in respiratory specimens from a total number of 335 admitted patients between August 2009 and July 2012.

b Clinical data were extracted from hospitalized patient records.

c Age (mean = 29.4, median = 22.3, range = 0–81).

d Only classified as immunocompromised when CD4^+^ cell count was lower than 350 cells per µl.

e Three patients used high doses of corticosteroids for an endocrine, respiratory or nephrological disease. Three patients had a primary immunodeficiency and 1 patient had a congenital syndrome. One patient was prematurely born.

f Two patients had been transferred to another hospital.

### Immunosuppressive treatment of ferrets

We investigated whether the observations on prolonged virus replication and emergence of antiviral resistance in immunocompromised patients could be mimicked in ferrets receiving a cocktail of immunosuppressive drugs similar to that administered to SOT patients (combination of mycophenolate mofetil (MMF), tacrolimus and predisolone). First, pharmacokinetics were studied following oral administration of ferrets (n = 4) given 20 mg/kg MMF, 1 mg/kg tacrolimus and 8 mg/kg prednisolone (Table 2 and Supporting information Figure S1). From the concentration over time profiles, peak (C_max_) levels for MPA, the active form of MMF, were 65±30 µg/mL with trough (C_12_) levels becoming undetectable after 8 hours. Peak and trough tacrolimus levels were 86±30 ng/mL and 14±30 ng/mL respectively. We determined area under the curve (AUC_0–12_) values of 54±14 µg·h/mL for MPA and 438±265 ng·h/mL for tacrolimus. In humans, MPA AUC_0–12_ values between 30–60 µg·h/mL and tacrolimus trough levels between 5–15 ng·h/mL are proposed [36], [37]. Because of a shorter MPA half life (*t_1/2_*) and high tacrolimus peak levels, we further optimized the ferret regime by administration of the cocktail every 12 hours containing half of the initial tacrolimus dose (0.5 mg/kg).

**Table 2:** Steady-state (day 4) pharmacokinetic parameters for mycophenolic acid, tacrolimus, oseltamivir phosphate and oseltamivir carboxylate in ferrets (n = 4).

	Dose (mg/kg)^a^	*T*_max_ (h)	*t_1/2_* (h)	*C_max_* (ng/mL)	*C_t = 12_* (ng/mL)	AUC_0–12_ (ng·h/mL)
**Immune suppressants**						
Mycophenolic acid (MPA)	20.0	0.33±0.18	0.73±0.36	65±20 (×10^3^)	<0.1	54±20^a^ (×10^3^)^b^
Tacrolimus	1.0^c^	0.79±0.58	3.60±2.29	86±30	14±14^d^	438±265
**Oseltamivir**						
Oseltamivir phosphate (OS)	10.0	1.00±0.71	3.22±0.51	2063±579	16±17	6491±1300
Oseltamivir carboxylate (OSC)		4.00±0.00	7.99±1.17	3052±448	833±247	20501±4005^e^

a Oral administration twice daily.

b Proposed human plasma levels (AUC_0–12_): 30–60 µg·h/mL [36].

c Dose adjusted to 0.5 mg/kg in the infection experiment.

d Proposed human whole blood trough levels (*C_t = 12_*): 5–15 ng/mL [37].

e Previously determined human plasma levels (AUC_0–12_): 3220±982 ng·h/mL (75 mg b.i.d.), 10100±2710 ng·h/mL (225 mg b.i.d.) and 19900±4840 ng·h/mL (450 mg b.i.d.) [53].

**Figure 2:**
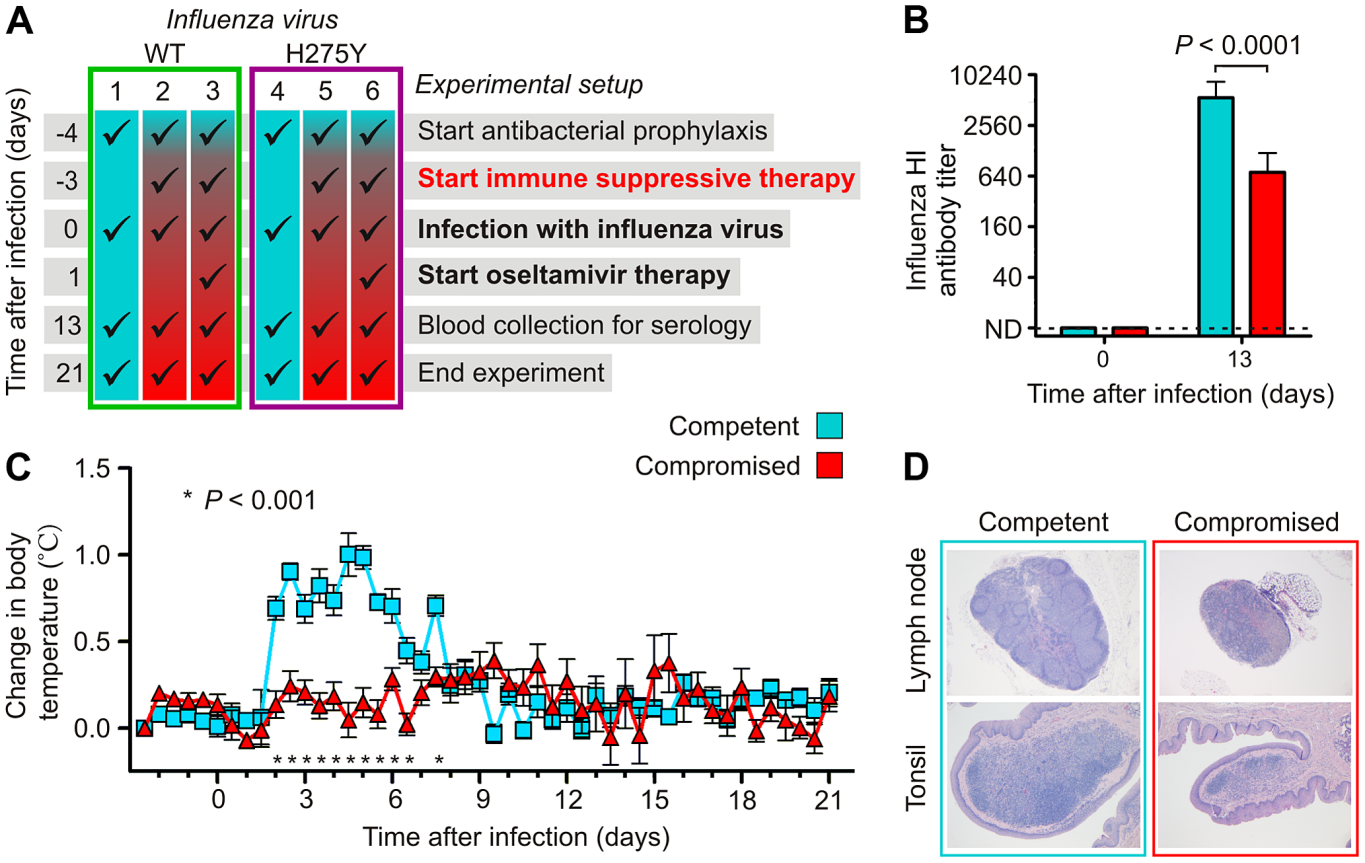
Ferrets on immune suppressive therapy show defective immune function. (**a**) Schematic of the experimental setup in which 6 groups of ferrets (n = 6) were infected on day 0 with wild type (WT; groups 1–3; green) or oseltamivir resistant (H275Y) mutant virus (groups 4–6; magenta). Three days before, immune suppressive therapy was started and added to the antibacterial cocktail of groups 2, 3, 5 and 6. Oseltamivir therapy was added to the drug regime of groups 3 and 6, 24 hours after infection. (**b**) Reduction of pH1N1 virus specific serum hemagglutination inhibiting (HI) antibody titers in the remaining (n = 14) immunocompromised ferrets compared to control (n = 12) ferrets. Data are mean ± s.e.m. The *P* value was calculated by Mann-Whitney U test. (**c**) Body temperature profiles show the absence of body temperature rise during the acute stage of infection in immunocompromised ferrets. Data are mean ± s.e.m. The *P* value was calculated by unpaired Student's *t* test. (**d**) Lymphoid tissues show deficient lymphoid follicle formation in lymph nodes and lymphocyte depletion in the tonsils of immunocompromised ferrets. Representative photomicrographs of tracheobronchial lymph nodes (original magnification 25×) and tonsils (original magnification 50×). Tissues were stained with hematoxylin and eosin (H&E).

Next, the effects of this cocktail on the ferret immune competence were studied. To this end, 6 groups of ferrets were inoculated intratracheally on day 0 with a wild type or H275Y mutant pH1N1 virus (Figure 2A). Of note, both viruses had been isolated by clonal culturing from the same respiratory sample taken from an immunocompromised patient on oseltamivir therapy [38]. Three days earlier, immunosuppressive therapy was started for all animals, except for the animals in control groups 1 and 4. On day 13, blood was collected and influenza antibody titers were determined in the ferret sera (Figure 2B). As compared to day 0, both the animals in the control groups (groups 1 and 4) and those on immunosuppressive therapy (groups 2, 3, 5 and 6), developed serum hemagglutination inhibiting (HI) antibody titers against the inoculated virus. However, antibody titers in the animals on immunosuppressive therapy were significantly lower (*P*<0.0001) (Figure 2B and S2A–D). As an indication of an activated immune response, body temperatures of all animals in the control groups raised from day 2, as compared to baseline, and remained higher until day 6 (Figure 2C and S2E–F). No significant change in body temperature was detected in the infected animals on immunosuppressive therapy, as is often seen in immunocompromised patients [39]. Finally, pathological examination of lymphoid tissues of animals sacrificed on day 21 revealed deficient lymphoid follicle formation in tracheobronchial lymph nodes and lymphocyte depletion in the tonsils of animals on immunosuppressive therapy (Figure 2D).

### Prolonged virus shedding in immunocompromised ferrets

**Figure 3:**
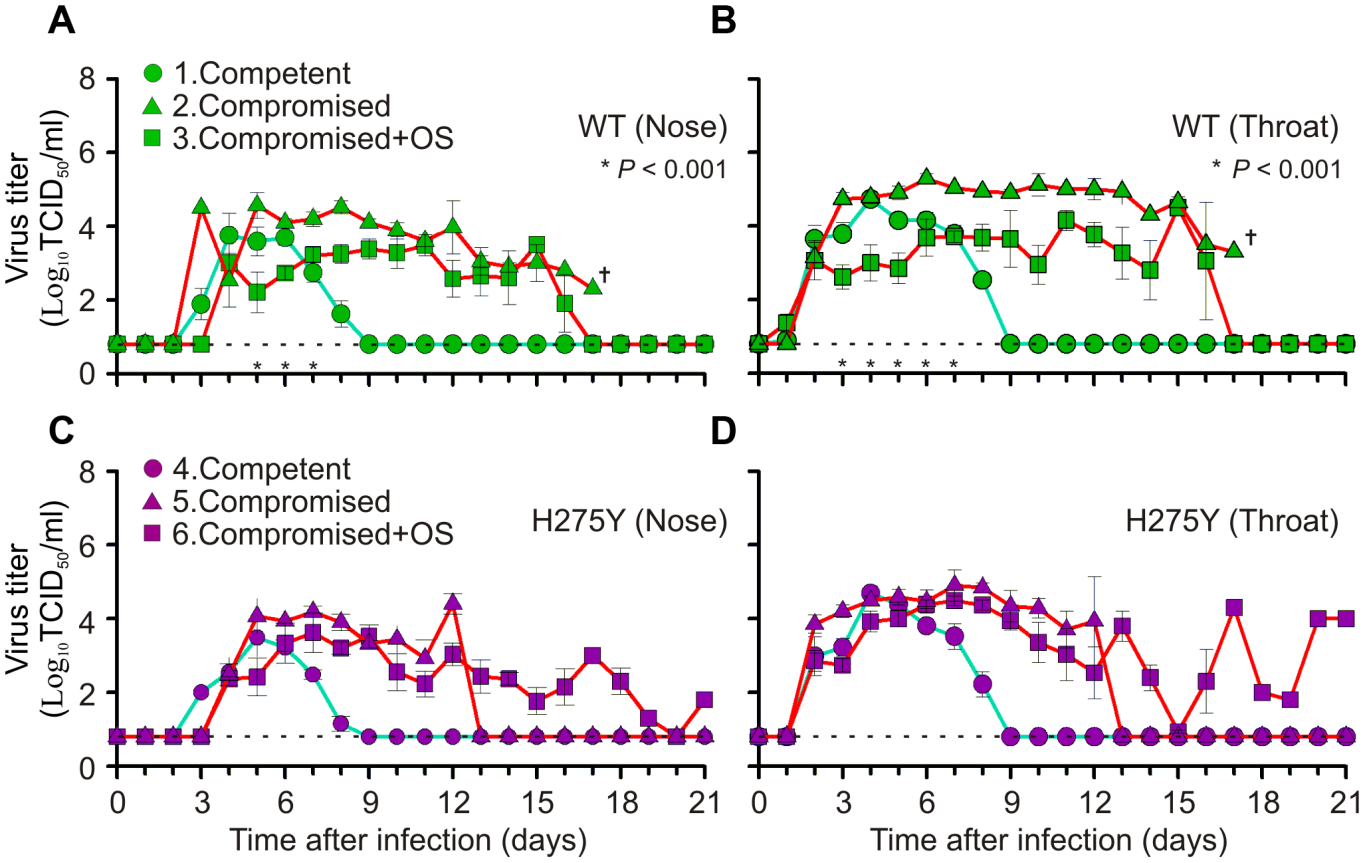
Prolonged virus replication in the upper respiratory tract of immunocompromised ferrets. Influenza virus titers were determined in nose and throat swabs daily taken from immunocompetent (blue lines) and immunocompromised ferrets (red lines). The animals were infected either with wild type (WT; green; **a**, **b**) or mutant virus (H275Y; magenta; **c**, **d**). The dotted line in each figure indicates the LLOD. Data are mean ± s.e.m. *P* values were calculated, until day 7, when all animals (n = 6) were still present in each group, by two-tailed Mann-Whitney U test comparing virus titers between untreated and oseltamivir (OS) treated immunocompromised ferret groups. By day 17, no ferrets were remaining in group 2.

The animals of groups 1, 2 and 3 had been inoculated with oseltamivir sensitive (wild type; H275) and the animals in groups 4, 5 and 6 with oseltamivir resistant virus (mutant; H275Y) (Figure 2A and 3). On day 2 post infection (p.i.), all 6 animals were found positive by virus culture from their throat (Figure 3 and S3). On day 3 p.i., virus was detected in the nose of five (group 1) wild type inoculated animals and one (group 4) animal inoculated with oseltamivir resistant virus (Figure S3A and C). In the animals infected with wild type virus, which were treated with oseltamivir (group 3), replication of virus in the nose was delayed as compared to the oseltamivir treated animals inoculated with mutant virus (group 6). By day 9, all animals in the control groups had cleared the virus. The surviving immunocompromised ferrets in the other groups were shedding virus for at least another 7 days, except for those in group 5. These animals had cleared the virus from the nose and throat by day 13 (Figure 3C and D).

### Oseltamivir therapy and emergence of oseltamivir resistance

In the animals of groups 3 and 6, oseltamivir therapy (10 mg/kg twice daily) was started 24 hours after infection and continued for 21 days (Table 2 and S2C). Until day 7, when still 6 animals were alive in each group, the viral loads in the oseltamivir treated animals infected with wild type virus was significantly lower on days 3, 4 and 5 in the nose and on days 3 to 7 in the throat (*P*<0.001). Such difference was not observed in the animals infected with oseltamivir resistant virus (Figure 3C and D).

**Figure 4:**
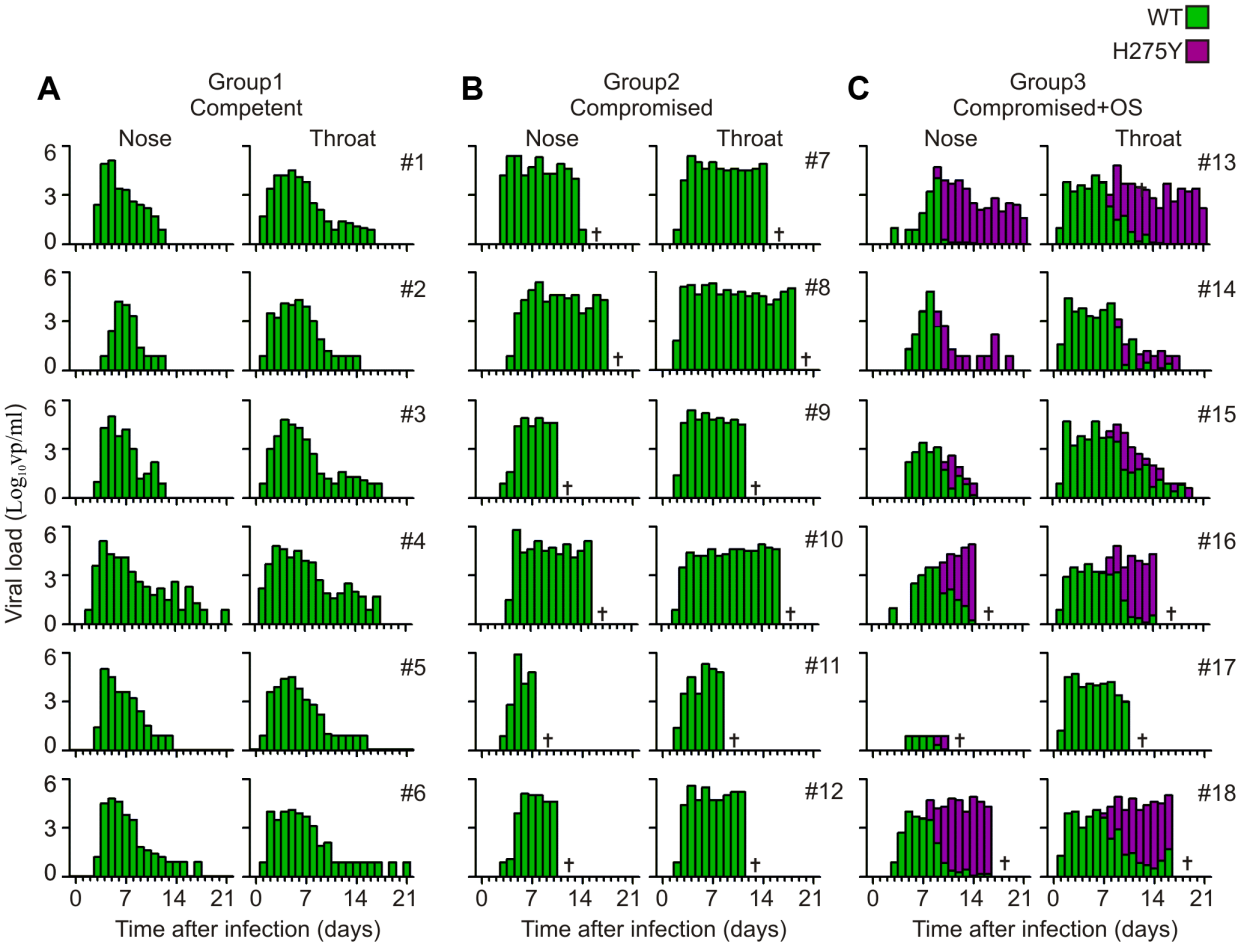
Emergence of oseltamivir resistance mutation H275Y in influenza virus quasispecies from ferrets infected with wild type virus. Viral RNA was detected using RT-qPCR in nose and throat swabs taken from immunocompetent (group 1; **a**) or immunocompromised ferrets (groups 2; **b** and 3; **c**). Ferrets in group 3 were treated with oseltamivir (OS). Bar colours indicate the absence (green) or presence (magenta) of the oseltamivir resistance mutation H275Y as detected by RT-PCR [40]. If both genotypes were detected in a sample, the proportion is stacked.

Emergence of oseltamivir resistance was studied in the animals infected with wild type virus (groups 1, 2 and 3) using an RT-PCR assay specifically detecting the H275Y oseltamivir resistance mutation (Figure 4) [40]. From day 8 onward, the H275Y mutation emerged in the virus population of all oseltamivir treated animals in both the nose and throat. The H275Y mutant became the major genotype 2 or 3 days later. No oseltamivir resistant viruses were detected in the immunocompromised wild-type virus infected animals that did not receive oseltamivir.

### Increased survival of immunocompromised animals treated with oseltamivir

**Figure 5:**
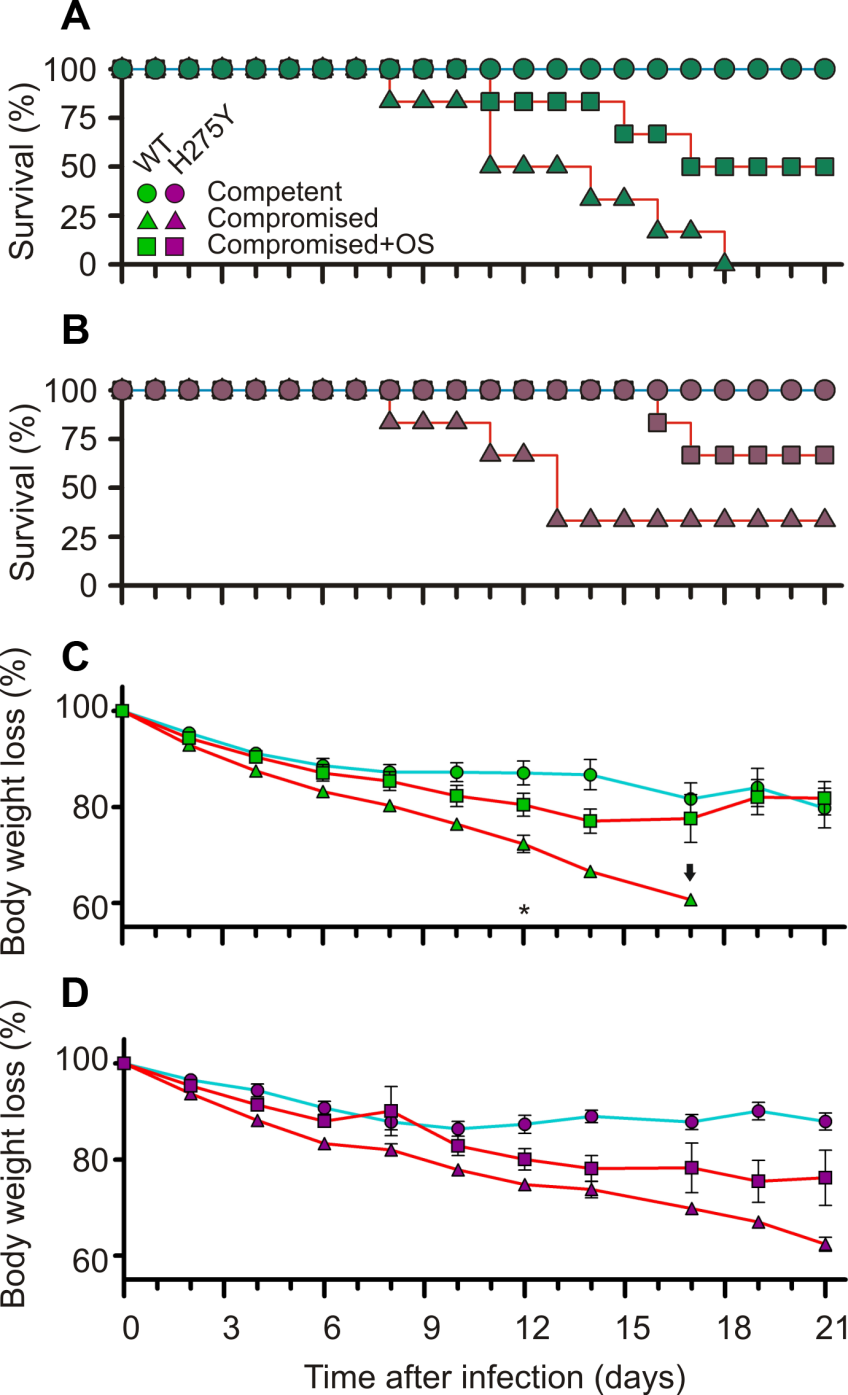
Increased survival and reduced loss of body weights of immunocompromised ferrets treated with oseltamivir. Kaplan-Meier survival curves for groups of immunocompetent ferrets (circles), immunocompromised ferrets (triangles) and immunocompromised ferrets treated with oseltamivir (squares) (**a;b**). Groups of ferrets were infected with wild type (green;**a;c**) or oseltamivir resistant (H275Y) mutant virus (magenta;**b;d**). Body weights are displayed from day 0 to day 21, with a two or three days interval (**c;d**). Data points represent mean ± s.e.m. of percentage body weight loss. Body weights at day 0 were set at 100%. The arrow indicates data point of only a single animal. The asterisk indicates significant difference between untreated and oseltamivir treated immunocompromised animals (*P* = 0.03). The *P* value was calculated by a two-tailed Mann-Whitney U test, when at least 3 animals were present in each group.

Unexpectedly, oseltamivir treatment appeared to have a positive effect on the proportion of surviving immunocompromised animals and animal body weight loss (Figure 5). Without oseltamivir treatment, half of the wild type infected group of animals had succumbed by day 11 and none of the remaining animals survived the complete 21 day experiment (0/6) (Figure 5A). However, when oseltamivir therapy was started 24 hours after infection, half of the animals were still alive at day 16 and these remaining animals all survived until day 21 (3/6). Of the immunocompromised ferrets, which were infected with the resistant virus (Figure 5B), half of the untreated animals had died before day 13, but two of the untreated animals and four of the treated animals survived the complete 21 day experiment.

In addition, oseltamivir treatment appeared to have a protective effect on body weight loss of the immunocompromised animals (Figure 5C and D). This trend was observed for both wild type and oseltamivir resistant virus infected groups, although statistical significant differences was found only for the wild type infected animals on day 12 when three animals were still alive (groups 2 versus 3; *P* = 0.03), and not for the oseltamivir resistant virus infected animals (groups 5 versus 6; *P* = 0.07).

## Discussion

Here we show that prolonged influenza virus shedding and the emergence of oseltamivir resistance are two phenomena commonly observed in immunocompromised patients during antiviral therapy. As resistance development is low (19 out of 874 (2.2%)) treated influenza virus infected patients [41], the incidence in immunocompromised patients appears to be considerably higher. Of all 71 immunocompromised patients infected with an influenza A virus in our hospital, at least 16 (23%) (11 pH1N1 and 5 H3N2) showed virus persistence for longer than 2 weeks with 5 of them harbouring oseltamivir resistant virus (5/16) (31%). Because 52% of the included patients were sent home before a virus negative follow-up sample had marked the end of infection, final conclusions on the true incidence of prolonged virus shedding and development of oseltamivir resistant virus cannot be made. However, this observation is in line with previously observed high resistance levels (33%) among paediatric cancer patients [35], and stresses the importance of a thorough evaluation of the currently used antiviral therapies in immunocompromised patients.

Since for both logistic and ethical reasons randomized studies are difficult to perform in often critically ill immunocompromised patients, we showed that prolonged influenza virus replication is also a common feature in immunocompromised ferrets. We observed an absence of a rise in body temperature, reduction of lymphocyte proliferation and follicle formation in ferret lymphoid tissues, which are also hallmarks in immunocompromised patients [42] and found a significant reduction of influenza virus specific antibodies in serum.

In the 2007/2008 H1N1 virus season an H275Y oseltamivir resistant mutant emerged, which had completely overtaken the circulating virus population by the end of 2008 [43]. This introduction of the H275Y mutation disqualified oseltamivir as the first line antiviral drug. It might happen again with pH1N1 virus if it would also harbour the H275Y mutation without loss of viral fitness. Recent reports on clusters of transmitted pH1N1 H275Y mutant viruses are the first indication that this may not be an unlikely scenario [44], [45]. Early studies on H275Y pH1N1 viral fitness were performed on viruses isolated shortly after the start of the 2009 pandemic. These viruses were found to be at least slightly compromised in their pathogenicity and replication capacity [46], [47], [48], [49]. We also used an early H275Y virus isolate in our experiment [38]. If virus replication is not tempered by adequate immune responses, duration of virus shedding will eventually be restricted by an exhaustion of susceptible host target cells. This then may explain why replication of this apparently less pathogenic virus lasted longest in our immunocompromised ferrets (Figure 3D). Priority should now be given to study the overall fitness of these recent pH1N1 viruses that appear not to be affected by the H275Y change. The use of our immunocompromised ferrets seems to be a very suitable strategy for this purpose, because subtle fitness costs may be amplified in these animals [50].

In our study, immunocompromised ferrets were treated with an oseltamivir dose of 10 mg/kg twice daily. This dose is equivalent to a much higher human dose than the currently recommended dose of 75 mg twice daily [51]. We observed that, for animals infected with either wild type or H275Y mutant virus, high dose oseltamivir treatment was still beneficial. Currently the World Health Organisation recommends switching to zanamivir when dealing with such a resistant virus [52]. However, in the light of our data, discontinuation of oseltamivir therapy may not always be the best strategy. An increased oseltamivir dose for the treatment of immunocompromised patients may be considered as a future antiviral strategy. However, obviously this will need further clinical investigation first. Of note, an increased dose of oseltamivir is well tolerated in humans [53]. We observed lower mortality in the treated animals infected with H275Y mutant virus without an observed difference in virus titers in the upper respiratory tract. In these ferrets, oseltamivir carboxylate plasma levels peaked (C_max_) from 3052 ng/ml in 4 hours to 833 ng/ml 12 hours after administration (Table 2 and Figure S1C). These levels were still about 100 and 30 times higher than the 50% inhibitor concentration of an H275Y mutant virus, which is roughly 30 ng/ml (∼100 nM) [54]. It is therefore plausible that oseltamivir therapy reduced H275Y mutant virus titer in the lungs, but not in the upper respiratory tract. This had been observed before and could explain the lower mortality rates in the treated animals [55]. It will therefore be of interest to study penetration of oseltamivir throughout the ferret respiratory tract in more detail, which may be expected to have a direct impact on the effectiveness of the presently recommended human dose in immunocompromised ferrets. In conclusion, both our clinical observations and ferret experiments show that viral clearance cannot be achieved in the immunocompromised host solely by the use of currently used antiviral therapy. Our immunocompromised ferrets may therefore be an excellent alternative to evaluate and explore novel therapeutic and immunization strategies for immunocompromised patients.

## Materials and Methods

### Patient inclusion criteria and diagnostics

We identified patients hospitalized in the Erasmus Medical Centre (ErasmusMC), a large (>40,000 admissions in 2011) tertiary university hospital in the Netherlands, with an influenza A virus positive respiratory specimen taken between August 2009 through July 2012. Patients had a prolonged virus infection if the virus could still be detected after 14 days. Virological data, patient immune status and administration of antiviral therapy were obtained by reviewing medical records. Immunosuppression was defined as any of the following: receipt of treatment for any cancer, the use of any immunosuppressive medication to prevent transplant rejection or for management of pulmonary or autoimmune conditions, premature birth and below gestational age or a diagnosis of AIDS. Influenza A virus and the H275Y oseltamivir resistance mutation were detected by reverse transcriptase RT-PCR assays. These assays have been described previously [40], [56]. Informed consent was waived because patient inclusion was performed retrospectively and data were anonymously stored as agreed by the hospital medical ethical board (MEC-2012-463)

### Viruses used in ferret experiment

Two biologically cloned pH1N1 influenza viruses were used in this study. Both viruses were isolated from an oseltamivir treated patient during the first wave of the pandemic in October 2009. Wild type influenza virus A/Netherlands/1715b/2009 (genbank ID code: CY065810) and H275Y mutant virus were isolated from the original quasispecies by co-cultivation of a respiratory sample in a Madin-Darby Canine Kidney (MDCK) cell culture in a single passage [38]. Biological clones were then obtained by 3 additional MDCK passages performed under limiting virus concentrations. As determined by full-genome Sanger sequencing, the mutant virus contained, additional to mutation H275Y in the neuraminidase, an L233M mutation in PB2 and a V541L mutation in HA.

### Ferrets

Animal were housed and experiments were conducted in strict compliance with European guidelines (EU directive on animal testing 86/609/EEC) and Dutch legislation (Experiments on Animals Act, 1997). The protocol was approved by the independent animal experimentation ethical review committee from the Netherlands Vaccine Institute (permit number 200900201). All experiments were performed under animal bio-safety level 3 conditions. Animal welfare was observed on a daily basis, and all animal handling was performed under light anaesthesia using a mixture of ketamine and medetomidine to minimize animal suffering. After handling atipamezole was administered to antagonize the effect of medetomidine. All ferrets were eleven-month-old purpose-bred males (body weights between 1562 and 2362 g) and were seronegative for Aleutian disease virus and circulating influenza virus (sub) types A/H1N1, A/H3N2 and B virus. The animals were maintained in standard housing and were transferred to negatively pressured glove boxed on the day immunosuppressive therapy was started. They were provided food *ad libidum* with commercial food pellets and water. Approximately three to four weeks prior to the experiment a temperature logger (DST micro-T ultra small temperature logger; Star-Oddi, Reykjavik, Iceland) was placed in the peritoneal cavity of the animals. This device recorded the body temperature of the animals every 10 minutes. From day −8 to day −4, an average baseline temperature was recorded for each group of 6 animals.

### Immunosuppressive, antibiotic and antiviral drugs

The following immunosuppressive drugs were used to suppress the immune system of ferrets: Mycophenolate mofetil (MMF) (CellCept, Roche, The Netherlands) powder for infusion, tacrolimus concentrate (5 mg/ml) for infusion (Prograft, Astellas Pharma BV, Leiderdorp, The Netherlands) and prednisolone sodium phosphate (5 mg/ml) oral solution (Hospital Pharmacy, UMCN St Radboud, Nijmegen, The Netherlands). All ferrets received an antibiotic prophylaxis of amoxicillin supplemented with 62.5 mg clavulanic acid (250/62.5 mg per 5 ml) oral suspension (Pharmachemie BV, Haarlem, The Netherlands). Prodrug oseltamivir phosphate, used in the ferret experiments, was kindly provided by Hoffman-La Roche LtD. (Tamiflu, Basel, Switzerland).

### Other chemicals and reagents

Oseltamivir standards for mass spectrometry, oseltamivir phosphate (OS), oseltamivir-d3 (OS-d3), oseltamivir carboxylate (OSC) and oseltamivir carboxylate-d3 (OSC-d3) were purchased from Toronto Research Chemicals (Toronto, Canada). Mycophenolic acid (MPA) standard and internal standards (MPAC) were purchased from Sigma Aldrich (Zwijndrecht, the Netherlands) and from Hoffman-La Roche LtD. respectively. The tacrolimus standards and internal standards (Ascomycin) were purchased from, respectively, Chromsystems and Sigma Aldrich. ULC/MS grade methanol and water containing 0.1% formic acid were obtained from Biosolve (Valkenswaard, the Netherlands). Trichloro acetic acid (TCA) and formic acid (>96%, HCOOH) were obtained from Sigma Aldrich and both were from ACS reagent quality.

### Administration of the drugs

A schematic of the ferret experiment is presented in Figure 2A. Shortly before gavage, drugs were prepared as follows: MMF was dissolved in a 5% glucose solution (Baxter, Unterschleisheim, Germany) to 33 mg/ml. Amoxicillin/clavulanic acid was diluted 5 times in water to obtain a suspension containing 50/12.5 mg/ml amoxicillin/clavulanic acid. Oseltamivir phosphate was dissolved in 5% glucose to 20 mg/ml. These intermediate preparations and the ready-to-use tacrolimus and prednisolone solutions were then used to dose the animals orally and twice daily, as follows: four days before infection, all 6 groups received 10/2.5 mg/kg amoxicillin/clavulanic acid diluted in 5% glucose to a final administration volume of 4 ml/kg. One day later, antibiotic prophylaxis was supplemented to the regimes of groups 2, 3, 5 and 6 with 20 mg/kg MMF, 0.5 mg/kg tacrolimus and 8 mg/kg prednisolone retaining the administration volume of 4 ml/kg. On day 1, 24 hours after infection, therapy of group 3 and 6 was further supplemented with 10 mg/kg oseltamivir phosphate for the remaining 20 days of the experiment. The dose of prednisolone was halved every 7 days from 8 mg/kg in the first to 1 mg/kg in the last week.

### Virology and serology

On day 0, three days after start of immunosuppressive therapy, ferrets were intratracheally infected with 1×10^4^ TCID_50_ of wild type (groups 1, 2 and 3) or mutant virus (groups 4, 5 and 6). Each day, pharyngeal and nasal swabs were collected just before administration of the drugs. Swabs were resuspended in 3 ml virus transport medium [57], and aliquots were made and used either directly for online detection of viral RNA by RT-PCR or stored at −80°C for retrospective virus titration. An electron microscopy counted influenza A virus stock was run in parallel to convert RT-PCR cycle threshold (CT) values into a viral particle count. Blood samples for serum and plasma were collected on day 13 after infection. Influenza antibody titers were determined as described previously [58].

### Oseltamivir blood plasma levels

Oseltamivir and MMF plasma levels and whole blood tacrolimus levels were determined in a pharmacokinetic pilot study. For 4 days, four groups of ferrets (n = 4) received MMF, tacrolimus, or oseltamivir in combination with amoxicillin/clavulanic acid and prednisolone or as the complete cocktail. On day 4, blood was collected from these animals after 0, 10, 20, 30 minutes and 1, 2, 4, 5, 8 and 12 hours after administration of the drugs in order to determine MMF and tacrolimus levels, as described previously [59, unpublished data]. Ferret oseltamivir plasma levels were determined as described previously with some modifications [60]. Calibrators used for the determination of the calibration curve of OS and OSC were prepared from one single stock solution in plasma each containing 50 µg/ml. Calibrators were then prepared by serial dilutions using drug-free plasma. Calibrators for OS and OSC yielded following concentrations: 5000, 1500, 750, 500, 250, 150, 50, 12.5, 2.5 and 0 ng/ml (blank). Aliquots of 50 µl were spiked with 5 µl internal standard solution containing 50 µg/ml OS-d3 and OSC-d3. To calibrators and ferret plasma (K_2_EDTA) samples, 5 µl of a 50% TCA solution (w/v) was added for plasma protein precipitation. Precipitated plasma proteins were removed by centrifugation for 10 minutes at 2000×g at ambient temperature. De-proteinized plasma samples (20 µl) were 2.5 times diluted with ultrapure water and 40 µl of the diluted samples were injected by an auto-sampler (kept at 4°C) into the liquid chromatography mass spectrometry (LC-MS) system. The calibration curves for OS and OSC showed a linear relationship between the SRM peak area of ratios between OS/OS-d3 and OSC/OSC-d3, respectively (OS; r^2^ = 0.9966 and OSC; r^2^ = 0.9970). The LLOQ and LOD were determined according to FDA guidelines and were, respectively, 2.5 and 1.0 ng/ml for both OS and OSC. The LC-MS system used was an 4000 API triple quadruple mass spectrometer containing a Turbo V electron spray ion source (ESI) (AB Sciex, Concord, Canada) operating in the positive ionization mode using selected reaction monitoring (SRM) in combination with a Dionex Ultimate 3000 UHPLC system (Amsterdam, the Netherlands) using an Ascentis Express RP-C18 column (100×2.1, 2.7 µm, Supelco, Munich) applying a gradient separation at 30°C.

### Pathology

Samples for histological examination of the tonsils and tracheobronchial lymph nodes were taken to evaluate the immune status and were stored in 10% neutral-buffered formalin. Subsequently, these were routinely processed and embedded in paraffin wax, sectioned at 4 µm and stained with haematoxylin and eosin (HE) for examination by light microscopy.

### Statistical analysis

Data are reported as mean ± standard error of the mean (s.e.m). The *P* values for comparison of influenza HI antibody titers in figure 2B, virus titers in figure 3 and body weight loss in figure 5C were calculated using Mann-Whitney U test only if at least three animals were remaining in each experimental group. *P*≤0.05 was considered significant.

## Supporting Information

Figure S1 Mean steady state (day 4) pharmacokinetics of MMF, tacrolimus and oseltamivir in ferrets. Twice daily, four ferrets were given a cocktail of antibacterial prophylaxis, immune suppressive therapy (**a; b**) and oseltamivir phosphate (**c**) for 4 days. On day 4, blood was collected after 0, 10, and 30 minutes and 1, 2, 4, 5, 8 and 12 hours after the final cocktail was administered. Plasma levels of the active form of mycophenolate mofetil (MMF), the metabolite mycophenolic acid (MPA) (**a**), whole blood tacrolimus levels (**b**), oseltamivir phosphate (black squares; **c**) and its metabolite oseltamivir carboxylate (grey squares; **c**) plasma levels were determined by mass spectrometry. Area under the curve (AUC_0–12_), peak (C_max_) and trough (C_12_) levels and half-life (*t*_1/2_) values are presented in table 2. Data are mean ± s.e.m..
(TIF)

Figure S2 Ferrets on immune-suppressive therapy show reduced antibody titers. Reduction of serum hemagglutination inhibiting (HI) antibody titers against pH1N1 A/NL/602/2009 virus (**a**) and more distant viruses A/sw/NL/25/80 (**b**), A/IT/1443/76 (**c**), and A/New Jersey/08/76 (**d**). Individual data points represent antibody titers for each animal and horizontal bars represent the mean titer per group. Body temperature profiles show the absence of fever during the acute stage of infection in ferrets infected with wild type (**e**) or mutant (**f**) virus. Animals in this experiment were either immunocompetent (circles), immunocompromised (triangles) or immunocompromised and oseltamivir treated (squares). Data are mean ± s.e.m. Data used for Figure 2B are marked with an asterisk.
(TIF)

Figure S3 Total number of animals positive for replication competent influenza virus in the upper respiratory tract. Influenza virus titers (TCID_50_/ml) were determined in nose and throat swabs daily taken from immunocompetent (blue bars; group 1 and 4) and immunocompromised ferrets (red bars; group 2, 3, 5 and 6). The animals were infected either with wild type (WT; **a**, **b**) or mutant virus (H275Y; **c**, **d**). Ferrets in groups 3 and 6 were treated with oseltamivir (10 mg/kg twice daily) starting 24 hours after inoculation.
(TIF)
