# Supplementary figures and images for: Prolonged Influenza Virus Shedding and Emergence of Antiviral Resistance in Immunocompromised Patients and Ferrets
Source: PLoS Pathog. 2013 May 23;9(5):e1003343. doi: 10.1371/journal.ppat.1003343 (PMC3662664; doi:10.1371/journal.ppat.1003343)

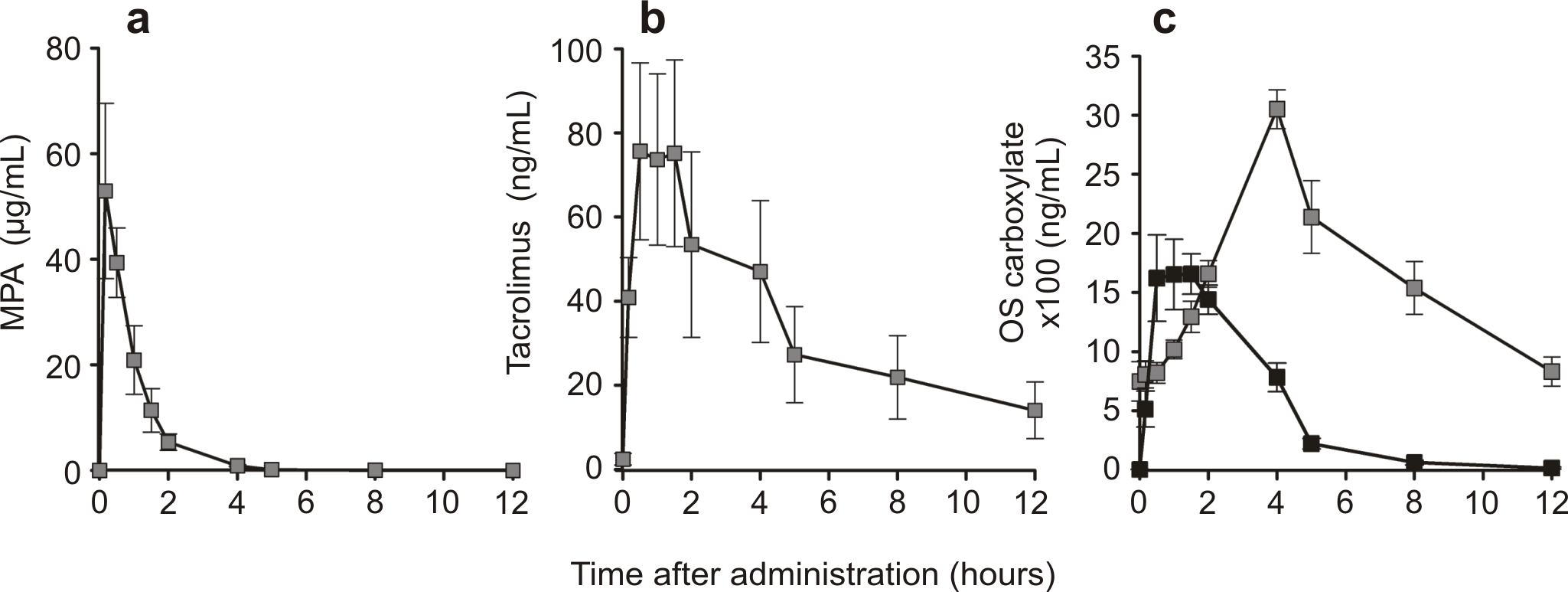

Supplement: Figure S1 — Mean steady state (day 4) pharmacokinetics of MMF, tacrolimus and oseltamivir in ferrets. Twice daily, four ferrets were given a cocktail of antibacterial prophylaxis, immune suppressive therapy (a; b) and oseltamivir phosphate (c) for 4 days. On day 4, blood was collected after 0, 10, and 30 minutes and 1, 2, 4, 5, 8 and 12 hours after the final cocktail was administered. Plasma levels of the active form of mycophenolate mofetil (MMF), the metabolite mycophenolic acid (MPA) (a), whole blood tacrolimus levels (b), oseltamivir phosphate (black squares; c) and its metabolite oseltamivir carboxylate (grey squares; c) plasma levels were determined by mass spectrometry. Area under the curve (AUC0–12), peak (Cmax) and trough (C12) levels and half-life (t 1/2) values are presented in table 2. Data are mean ± s.e.m.. (TIF) [file ppat.1003343.s001.tif]

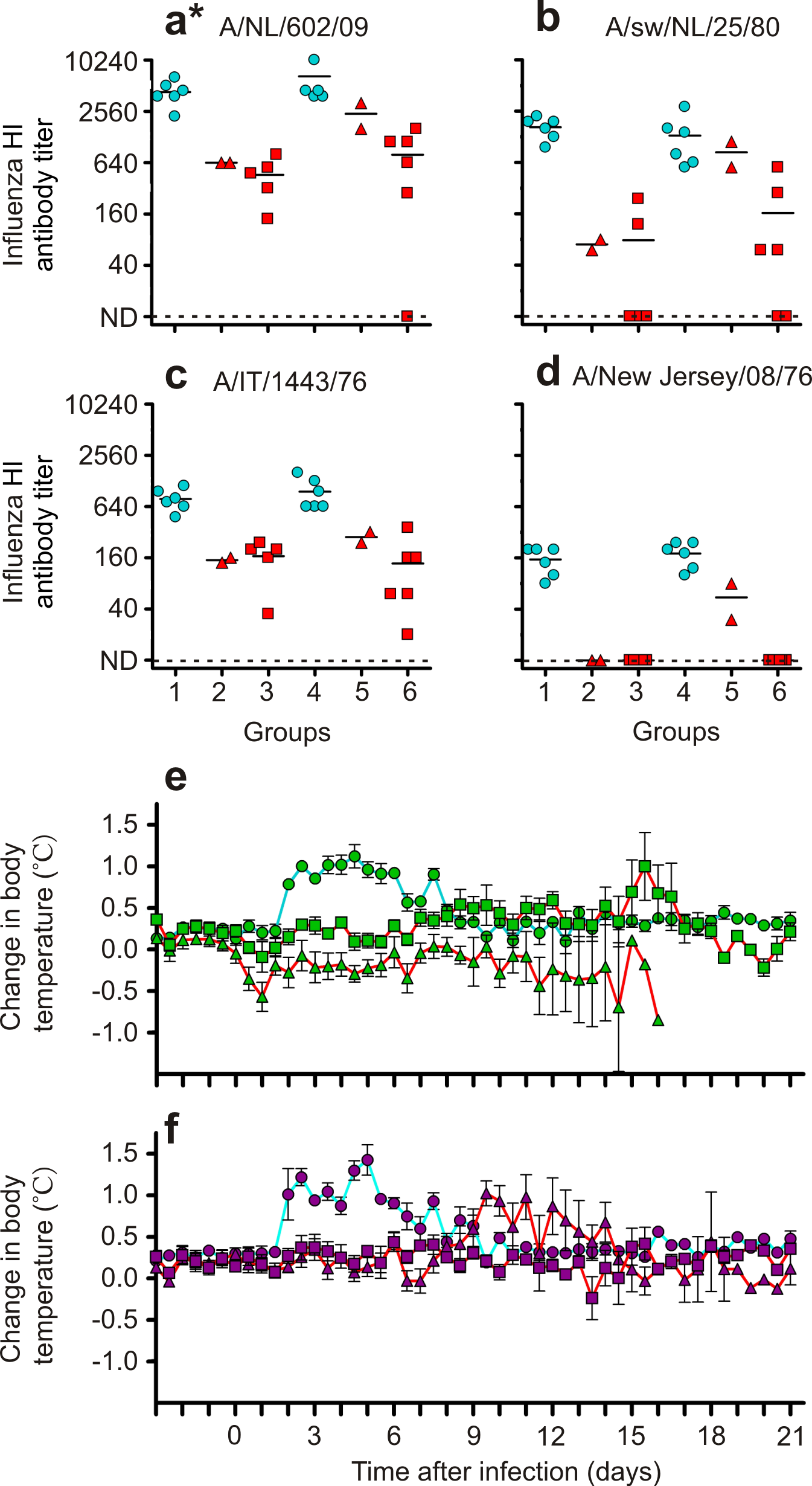

Supplement: Figure S2 — Ferrets on immune-suppressive therapy show reduced antibody titers. Reduction of serum hemagglutination inhibiting (HI) antibody titers against pH1N1 A/NL/602/2009 virus (a) and more distant viruses A/sw/NL/25/80 (b), A/IT/1443/76 (c), and A/New Jersey/08/76 (d). Individual data points represent antibody titers for each animal and horizontal bars represent the mean titer per group. Body temperature profiles show the absence of fever during the acute stage of infection in ferrets infected with wild type (e) or mutant (f) virus. Animals in this experiment were either immunocompetent (circles), immunocompromised (triangles) or immunocompromised and oseltamivir treated (squares). Data are mean ± s.e.m. Data used for Figure 2B are marked with an asterisk. (TIF) [file ppat.1003343.s002.tif]

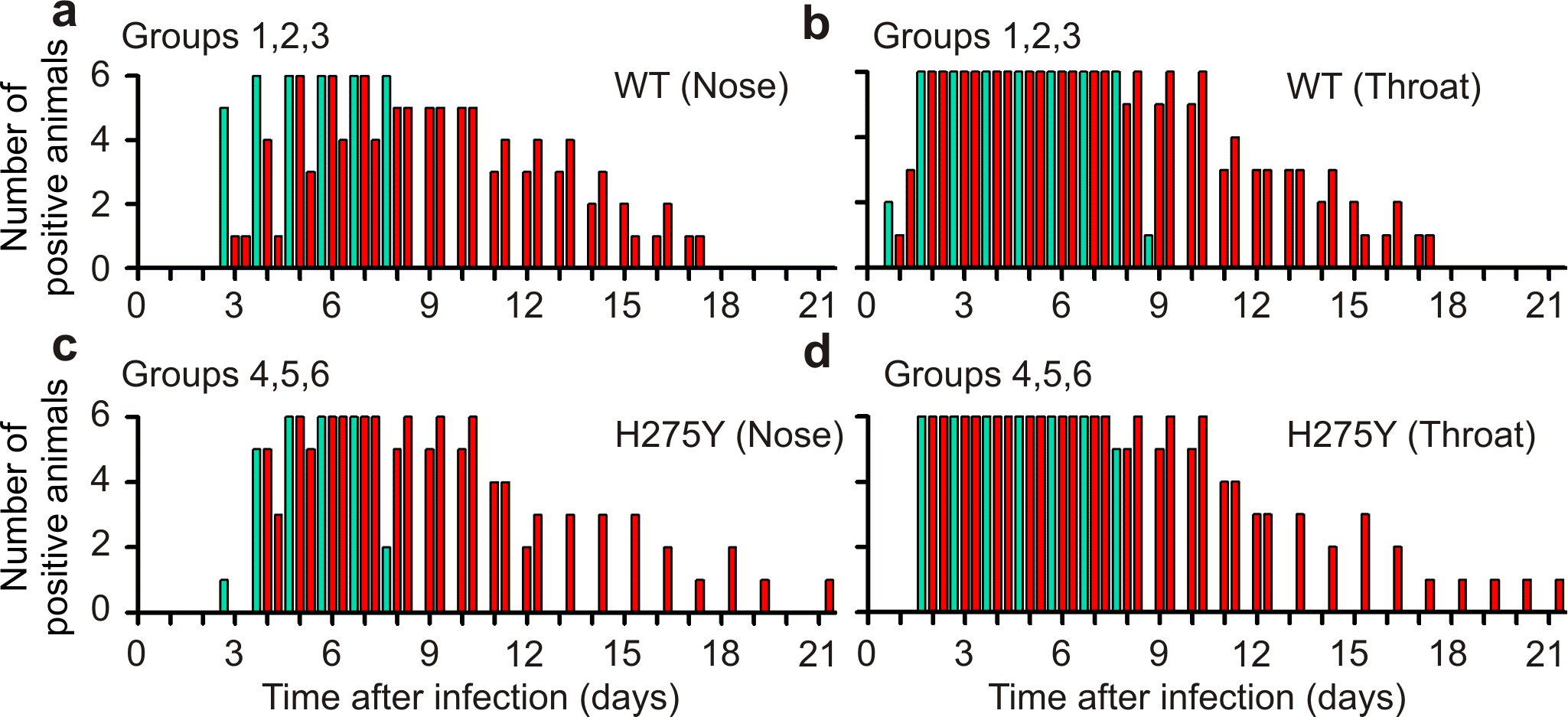

Supplement: Figure S3 — Total number of animals positive for replication competent influenza virus in the upper respiratory tract. Influenza virus titers (TCID50/ml) were determined in nose and throat swabs daily taken from immunocompetent (blue bars; group 1 and 4) and immunocompromised ferrets (red bars; group 2, 3, 5 and 6). The animals were infected either with wild type (WT; a, b) or mutant virus (H275Y; c, d). Ferrets in groups 3 and 6 were treated with oseltamivir (10 mg/kg twice daily) starting 24 hours after inoculation. (TIF) [file ppat.1003343.s003.tif]
